# Early Healing Events around Titanium Implant Devices with Different Surface Microtopography: A Pilot Study in an *In Vivo* Rabbit Model

**DOI:** 10.1100/2012/349842

**Published:** 2012-04-01

**Authors:** Ester Orsini, Stefano Salgarello, Désirée Martini, Beatrice Bacchelli, Marilisa Quaranta, Luciano Pisoni, Emma Bellei, Monika Joechler, Vittoria Ottani

**Affiliations:** ^1^Department of Human Anatomical Sciences and Physiopathology of Locomotor Apparatus, Human Anatomy Section, University of Bologna, via Irnerio 48, 40126 Bologna, Italy; ^2^Dental School, University of Brescia, Piazza Spedali Civili 1, 25123 Brescia, Italy; ^3^Department of Veterinary Medical Sciences, Faculty of Veterinary Medicine, University of Bologna, via Tolara di Sopra 50, 40064 Ozzano dell'Emilia, Bologna, Italy

## Abstract

In the present pilot study, the authors morphologically investigated sandblasted, acid-etched surfaces (SLA) at very early experimental times. The tested devices were titanium plate-like implants with flattened wide lateral sides and jagged narrow sides. Because of these implant shape and placement site, the device gained a firm mechanical stability but the largest portion of the implant surface lacked direct contact with host bone and faced a wide peri-implant space rich in marrow tissue, intentionally created in order to study the interfacial interaction between metal surface and biological microenvironment. The insertion of titanium devices into the proximal tibia elicited a sequence of healing events. Newly formed bone proceeded through an early distance osteogenesis, common to both surfaces, and a delayed contact osteogenesis which seemed to follow different patterns at the two surfaces. In fact, SLA devices showed a more osteoconductive behavior retaining a less dense blood clot, which might be earlier and more easily replaced, and leading to a surface-conditioning layer which promotes osteogenic cell differentiation and appositional new bone deposition at the titanium surface. This model system is expected to provide a starting point for further investigations which clarify the early cellular and biomolecular events occurring at the metal surface.

## 1. Introduction

Osseointegration is regarded as a fundamental criterion for long-term success of endosseous dental implants [1, 2]. It is based on the establishment of a primary mechanical stability and subsequent biological fixation [3]. Numerous factors may influence the rate of osseointegration and among them, the original bone architecture/density and the surface topography are likely to play a major role [4, 5].

Tooth replacement treatment, especially in the posterior region of the maxilla, generally implies placing implants into a bony bed characterized by a very thin cortical bone shell, an alveolar bone volume reduced in height, and an overall low bone density. The poor bone structural and architectural properties are accountable for a lower primary stability and the consequent higher failure rates claimed by clinical studies [6]. To date, many approaches have been tried to improve these clinical results, shortening healing periods and reducing loading time.

Implant topography modification treatments have provided surface microroughness in the range of 1–10 *μ*m which has shown to result in greater bone-to-implant contact area, greater biomechanical interlocking of the implant with host bone (i.e., mechanical stability), and enhanced osteoconductive features (i.e., early biological fixation) compared to implants with smooth surfaces [7, 8].

Actually, bone healing around dental implants is considered a pathway similar to that occurring after injury, based on the following successive phases: inflammation and necrosis, blood clotting organization and replacement, chemotaxis of pluripotential mesenchymal cells into the peri-implant site and adhesion on implant surface, neoangiogenesis, as well as differentiation of these cells into osteoclasts and osteoblasts which collaborate within temporary anatomic structures, named basic multicellular units (BMUs), by coupling bone resorption and new formation, thus leading to regeneration and remodeling [9].

It is well known that surface topography plays a role from the very beginning of these events; in particular, smooth surfaces integrate through appositional bone growth while microrough surfaces may show bone growth directly to the implant surface, according to a “contact osteogenesis” pattern [10]. There are clear experimental evidences that these surfaces do provide favorable conditions for attaching and stabilizing the blood clot elements which in turn will constitute a conditioning layer for cell interactions [11, 12]. The original surface conditioned by surrounded biological microenvironment can thus elicit subsequent events facilitating migrating osteogenetic cells to adhere to the implant surface, to differentiate, and to secrete biomolecules favoring earlier new bone formation [13].

Although many data have been published to date and despite the frequent clinical use of osseointegrated titanium implants, failures still occur, in many cases at early stages after insertion and apparently not related to surgical technique [14]. This decreased clinical predictability indicates that the bone healing around a titanium implant is a complex biological process not yet fully understood and underlines the necessity to clarify the initial responses of the surrounding tissue to implant surface. To our knowledge only a few *in vivo* studies have investigated the initial events taking place in the host tissues and at the interface with such implants.

The aim of the current pilot study was therefore to investigate the early healing events that were triggered by microrough surface implants inserted in trabecular bone over the period 0–6 days, combining surface characterization techniques with morphological and histochemical approaches. The authors also intended to establish a titanium device implantation model in trabecular bone to be used for further *in vivo* analysis of the biological responses evoked by microroughness of implant surface.

## 2. Materials and Methods

### 2.1. Implant Design and Surface Characterization

**Figure 1:**
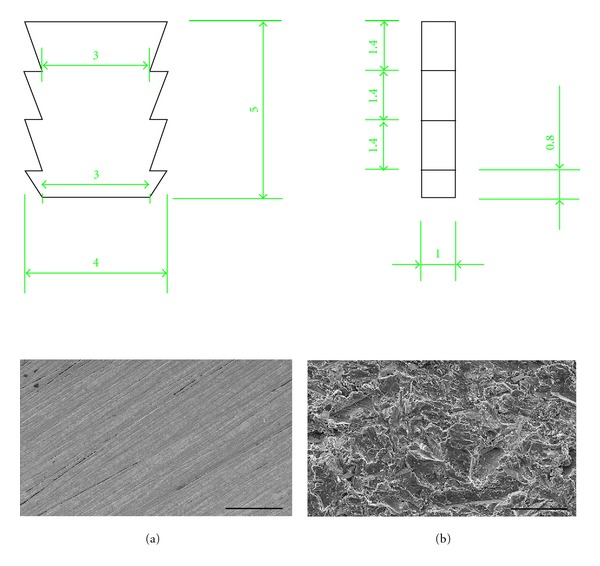
Top. Technical drawing of the tested implant devices. Bottom. SEM images of MS (a) and SLA (b) surfaces. Bar = 50 *μ*m.

Experimental implants consisted of custom-made, rectangular, titanium plate-like implants with flattened wide lateral sides, and jagged narrow sides. They were made of commercially pure titanium (grade IV-ISO 5832-2, CpTi) by Or-Vit (Castelmaggiore, Bologna, Italy). They measured 5 mm in length, 4 mm in width, and 1 mm in thickness (Figure 1). This design offered some advantages: (1) two flat surfaces for interaction with the biological microenvironment establishing a geometrically regular compartment once inserted in the animal model; (2) two jagged narrow edges ensuring a firm mechanical stability in the surgically created osteotomy; (3) a quite wide gap between the titanium surfaces and the osteotomy walls preventing the risk of rubbing against the osteotomy wall and damaging the titanium-bone interface when extracting the device; (4) an overall shape allowing to analyze early *in vivo* bone-implant retention and peri-implant microenvironment attached to the extracted implants overlooking the parameter “implant design” which actually plays a key role in low-density bone [15].

 Two kinds of surface textures were utilized: test and control. Test surfaces (SLA) were sandblasted, and acid-etched titanium plates resulting from a blasting treatment (with powders removing particles from the surface creating pits) followed by a chemical etching (with acids smoothing out some sharp peaks and removing chemical remnants), according to a registered process of Or-Vit, thus formed microroughened surfaces. Such surface exhibited an irregular profile with moderately deep pits alternated with metal peaks. The microroughness was found to be perceived by *in vitro* cells which resulted positively influenced in growth and metabolic activity [16] and to increase peri-implant osteogenesis *in vivo* [17]. Machined surfaces (MSs) obtained by a machining process were used as control (Figures 1(a) and 1(b)).

Implants were degreased in trichloroethylene, ultrasonically cleaned of contaminants in absolute ethanol, and supplied in individual gamma-sterilized surgical packs that were opened just before their surgical insertion in bone.

Surface characterization was performed according to a standardized protocol previously presented [16]. Briefly, the microtopography was qualitatively analyzed by means of scanning electron microscopy (SEM, Philips 515, Philips, Eindhoven, The Netherlands). Surface characterization in terms of roughness profile was performed under a roughness tester (Surfcom130A, Tokio Seimitsu).

### 2.2. Animal Model and Surgical Procedure

All surgical procedures were performed according to European and Italian legislation on animal experimentation and the ethical principles stated in the ‘‘Guide for the Care and Use of Laboratory Animals” (http://www.nap.edu/catalog.php?record_id=12910). The experimental protocol was submitted and approved by the Scientific Technical Committee and Ethical Committee of Alma Mater Studiorum-Bologna University under the Prot.58897-X10 All.22-11/21/2008–Rif BQ/mc/af. Twelve 8-month-old lop-eared New Zealand White female rabbits of 4.5 ± 0.5 kg in body weight (Farm Greci Agnese Bologna, Italy) were used. The proximal tibial epiphysis of rabbit was selected as the experimental animal model because of its anatomical heterogeneity which is regarded as a representative model for the jaw bony architecture [18]. The animals were housed in individual cages for a quarantine period of 7 days under the same environmental conditions (22°C ± 1; 55 RH ± 5%) and constant veterinary care, and they were fed with a standard diet and filtered tap water *ad libitum*.

After a preoxygenation period in an Oxygen cage, general anesthesia was induced with an intramuscular injection of Medetomidine (0.2 mg/kg-Domitor), Ketamine (10 mg/kg-Imalgene 1000), and Butorphanol (0.5 mg/kg-Nargesic) and maintained by a facial mask and an open circuit (T-Ayre modified Jackson Rees), with Isofluorane (1.5–2%) in O_2_. During the entire procedure, the rabbits were connected to a multiparametric monitor (Datex-Ohmeda S/5TM) and heated by means of a heated mat (AP 1137/FA, Dyaset S.r.l).

**Figure 2:**
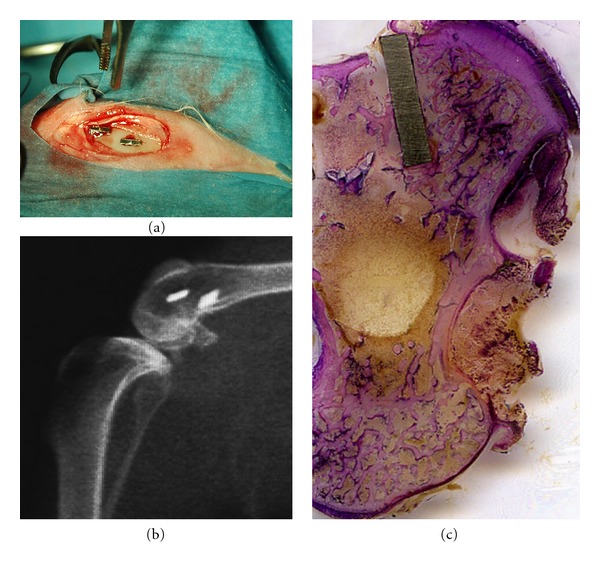
Clinical view of the device placement in rabbit proximal tibial epiphysis (a). X-ray micrograph of the surgical site (b). Histological ground section of an implant device. The implant is medially and laterally surrounded by cortical and trabecular host bone. LM, toluidine blue, and acid fuchsin staining, 1x.

The rabbit proximal tibial region was shaved and disinfected with Betadine prior to surgery. The medial aspect of each epiphysis was surgically exposed via a skin incision and the muscles were dissected to allow sterile elevation of the periosteum. Tibial epiphysis was drilled at low speed and under a profuse irrigation with cold sterile 0.9% NaCl solution maintained throughout the process to prevent bone necrosis. In order to gain access to the deep trabecular bone compartment and ensure the device to the thin external cortical layer, two slightly undersized holes, 3.9 mm diameter and 1.2–1.5 cm distance apart, were drilled in each epiphysis. Two implant devices, one for each surface type, were press-fit tightened into the surgically prepared holes in an alternating pattern (Figure 2(a)). After the implants were seated, surgical sites were closed in layers by suturing muscles, fascia, internal dermal layer, and outer dermis. The implants were radiographically located (Figure 2(b)). During the postoperative period, antibiotic (Enrofloxacin Baytril 10 mg/kg SC sid) and analgesic (Carprofen Rymadil 2 mg/kg SC SID) therapies were administered and the rabbits were housed in single boxes under the same environmental conditions. Animals had water and were fed *ad libitum* while healing. The animals were randomly divided into three groups of four animals each, according to the experimental times of 4 hours (representing the steady-state time or day 0), 3 and 6 days after implantation. At the end of each experimental time, the animals were resubmitted to surgery under general anesthesia in order to extract some implant devices and then pharmacologically euthanized with an intracardiac injection of Tanax (Embutramide mebenzonium iodide-tetracaine hydrochloride; 0.2 mL/kg). The proximal tibial regions were stripped of soft tissue. Each implant was surgically exposed via sharp dissection. The proximal tibial regions were excised with an oscillating saw and the block specimens including the implants were isolated. The entire blocks and the isolated titanium plates were processed for histological and ultrastructural evaluations.

### 2.3. Histological, Ultrastructural, and Histochemical Analyses

Most retrieved implant surrounded by nondecalcified cylindrical bone segments were immediately fixed by immersion in 10% buffered formalin solution (pH 7.2), dehydrated in an ascending series of ethanol concentrations, cleared in xylene and subsequently embedded in a methacrylate resin (Technovit 9100 New Heraeus Kulzer GmbH, Germany) which was polymerized at −10°C avoiding negative side effects of a heat-polymerizing resin. The embedded samples were glued with acrylic resin (EXAKT Technovit 7210 VLC Adhesive, Heraus Kulzer, Wehrheim, Germany) to plexiglas slides and cut longitudinally, parallel to the long axis of the implant, resulting in 150 *μ*m thick sections. These sections were grounded to a final thickness of about 50 *μ*m (MT MICROMET, Saw and Grinding System, Remet, Bologna, Italy) and superficially stained with toluidine blue and acid fuchsin for general morphological analysis. Examination was performed under a Leitz Orthoplan light microscope (LM, Leica Microsystem Inc., Bannockburn, Ill, USA) equipped with a Leica camera image acquiring system. Some embedded sections were processed for histochemical demonstration of alkaline phosphatase (ALP) and TRAPase (tartrate-resistant acid phosphatase) activities. These methods involved deacrylation of the resin sections in 2-methoxyethylacetate and rehydration up to 0.1 M phosphate buffer. Briefly, for ALP activity, naphthol-AS-TR-phosphate was used as a substrate and the coupling salt reaction was carried out with Fast blue RR salt (code 30-30121LY, Bio-Optica S.p.A., Milan, Italy). The incubation was carried out for 120 minutes at 37°C. TRAPase activity was detected by the azodye method with slight modifications. The incubation medium comprised 0.06% fast red violet LB salt, 0.01% naphtol AS-MX phosphate (Sigma chemical Co., St. Luis, Mo), and 50 mM L-(+) sodium tartrate in 0.1 M acetate buffer pH 5.2. The incubation was carried out for 60 minutes at 37°C.

Some unstained methylmethacrylate-embedded sections were also processed for ultrastructural investigations, according to a standardized protocol which set a pretreatment step in order to remove the resin [19]. Briefly, the sections were deacrylated using (2-methoxyethyl)-acetate (Carlo Erba, Milan, Italy), renewing the solution every 2 days and periodically checking on the deacrylation process. They were then rinsed to remove (2-methoxyethyl)-acetate by immersion in 100% ethanol, immersed in hexamethyldisilazane (Sigma-Aldrich, Seelze, Germany), and dried under a hood. Dried sections were mounted on aluminum stubs using a carbon bioadhesive film and coated with gold/palladium for SEM observation using a secondary electron probe at a voltage of 15 kV.

Some retrieved isolated titanium plates were processed for SEM investigations. The isolated plates were gently rinsed with PBS containing Ca^2+^ and Mg^2+^ to prevent detachment of interface from the surface. Cells were fixed with Karnowsky solution (glutaraldehyde 1.5% paraformaldehyde 1% in cacodylate buffer) for 10 minutes then rinsed 3 times with 0.1% cacodylate buffer, postfixed for 20 minutes with 1% Os_2_O_4_ in cacodylate buffer, dehydrated with ethanol, and eventually treated with hexamethyldisilazane for 10 minutes. The samples were observed under a scanning electron microscope (Philips 515 Scanning Electron Microscope, Philips, Eindhoven, The Netherlands).

## 3. Results

### 3.1. Surface Characterization

Roughness investigation underlined the differences between the two surfaces in terms of surface profile parameters. In particular, the MS sample exhibited the lowest values (*R*_*a*_ 0.25 *μ*m, *R*_*t*_ 2.51 *μ*m) of rugosity compared to the SLA surface (*R*_*a*_ 1.11 *μ*m, *R*_*t*_ 9.01 *μ*m). The parameter *R*_*q*_, which expressed the irregularities distribution, was slightly higher for the SLA surface compared to MS (1.63 *μ*m versus 0.69 *μ*m). These data demonstrated that the treatment created pits and peaks the additional etching smoothens out some sharp peaks and the SLA surface exhibited a regular and quite homogeneous concave profile superimposed over the grooves. SEM analysis confirmed the two substantially different types of topography. The MS surface exhibited a smooth appearance with oriented and parallel grooves in the l–10 um range as a turning manufacturing process feature. SLA surfaces, on the other hand, showed a rough profile characterized by small irregular cavities homogeneously alternated with metal peaks (Figures 1(a) and 1(b)).

### 3.2. Histological, Histochemical, and Ultrastructural Analyses

All experimental animals survived and recovered quickly after implant placement without any clinical or postoperative complications. The healing was uneventful, with the initial postimplantation inflammation subsiding rapidly in 2 days.

All implants were *in situ* when animals were euthanized. At retrieval, a macroscopic evaluation of the implant site was performed, which confirmed that all the devices had been correctly inserted and no signs of infection were observed. Each histological ground section comprised the implant device and the surrounding host bone. Only the most proximal portion of the implant was held in position by the thin superficial cortical bone layer (ensuring a firm mechanical stability) while the remaining subcortical portion protruded into the deep trabecular bone network (Figure 2(c)).

#### 3.2.1. Four Hours (Day 0, Steady State)

**Figure 3:**
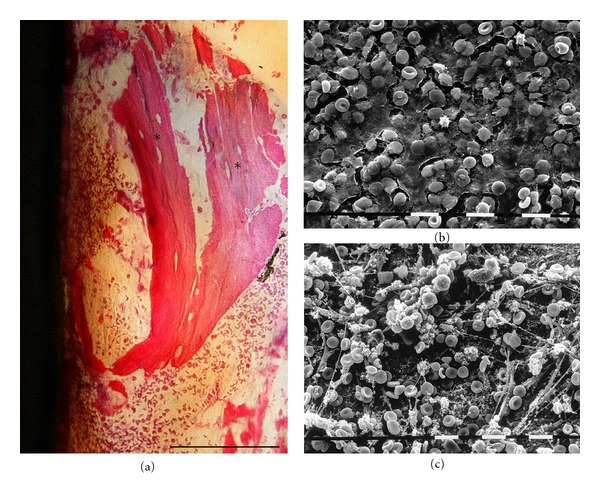
Day 0. Histology of longitudinal ground section (a). Empty osteocytic lacunae (*). LM, toluidine blue, and acid fuchsin staining. Bar = 100 *μ*m. SEM images of MS (b) and SLA (c) surfaces extracted at 4 hours after insertion. Bar = 10 *μ*m.

Four hours after implant placement (day 0), the ground section observed under light microscope showed a quite wide gap area (1.3 mm ± 0.2 mm SD) between the wall of the surgically created osteotomy and the titanium device. A coagulum of blood cells entrapped in a thin network of fibrin filled this gap reaching the implant surface. A notable inflammatory cell infiltration, mainly neutrophilic granulocytes, along with primitive marrow cells and degenerating cellular elements was also observed throughout the interface of the bone and the implant cavity. Several chips of cortical and spongy host bone, shattered by drilling and placing procedures and surrounded by blood elements, could be seen not only adjacent to the implant surface but also quite far from the implant cavity (Figure 3(a)). Traumatized preexisting bone trabeculae near the implant cavity, as well as host bone chips, showed surface areas characterized by empty osteocytic lacunae, as documented by the lack of the typical deep blue staining of the osteocytic nuclei. The ultrastructural observation of extracted device surfaces under SEM confirmed the histological analysis. These surfaces were covered by a network of fibrin entrapping a large number of cells identified through their morphology: biconcave erythrocytes, platelets, neutrophils, and macrophages. This blood clot showed a different affinity for the two surfaces, being more thick and adherent on machined surfaces compared to SLA. Actually, on MS, a mesh of fibrin-like fiber bundles arranged on the superficial grooves and entrapping numerous cells could be seen (Figures 3(b) and 3(c)).

#### 3.2.2. Three Days

**Figure 4:**
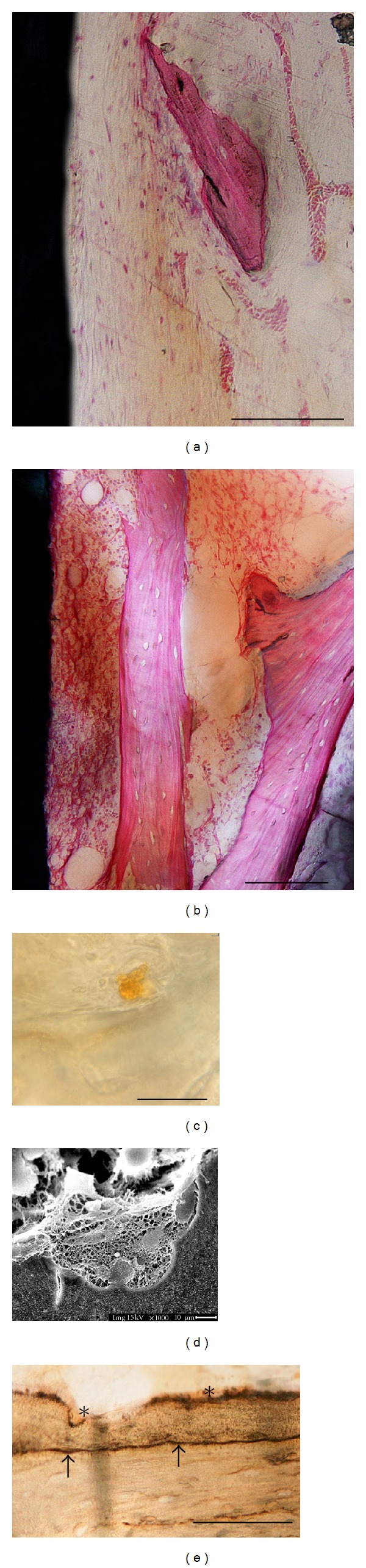
Day 3. Histology of longitudinal ground sections of MS (a) and SLA (b) surfaces. LM, toluidine blue, and acid fuchsin staining. Bar = 100 *μ*m. TRAP staining of osteoclast at the trabecular bone surface (c). Bar = 100 *μ*m. SEM images of resorption lacunae at the trabecular bone surface (d). ALP staining of osteoid rim at the trabecular bone surface (e). ALP positivity for bone areas facing the marrow cavity (*); ALP positivity for the incremental line in close connection to the old bone (arrows). Bar = 100 *μ*m.

After a three-day healing period, the general histological appearance was similar to the steady-state samples. The original hole that was drilled to place the implant could still be recognized. The elements of the primitive blood clot were still present as well, especially in regions close to the device, but they were undergoing a wound-cleansing process. In particular, the inflammatory cell infiltration showed a tendency to disappear in the tissue around the implant, many multinucleated giant cells occurred, and portions of the coagulum were replaced with a healing tissue containing undifferentiated fibroblast-like mesenchymal cells and new vascular structures. Observation of ground sections allowed the appreciation of a loose connective stroma, poorly vascolarized and organized, with elongated fibroblast-like cells at the MS interface (Figure 4(a)). On the contrary, at SLA device, interface cells formed a more consistent layer along the surface of the implant. These cells showed a polygonal shape and numerous blood vessels appeared near the implant and surrounded by cells (Figure 4(b)). Trabeculae showing areas with empty osteocytic lacunae were still visible after three days. On the surface of these bony structures, signs of bone resorption were observed, as indicated by the presence of scalloped resorption areas located at the site facing the bone marrow. Histochemical TRAPase staining showed intense reactivity for these cells (Figure 4(c)) and SEM observation confirmed the histological data (Figure 4(d)). Initial osteoid deposition was detected next to these resorption regions. The bony surface was lined by cuboidal osteoblast-like cells that were characterized by a pronounced deposition activity (extracellular matrix), as histologically shown by intense stainability (Figure 4(b)). Histochemical analysis of the enzyme activities supported the morphological data. A positivity for ALP (resulting in a dark stained osteoid rim) was detected at some bone areas facing the marrow cavity and at the incremental line in close connection to the old bone (Figure 4(e)).

#### 3.2.3. Six Days

**Figure 5:**
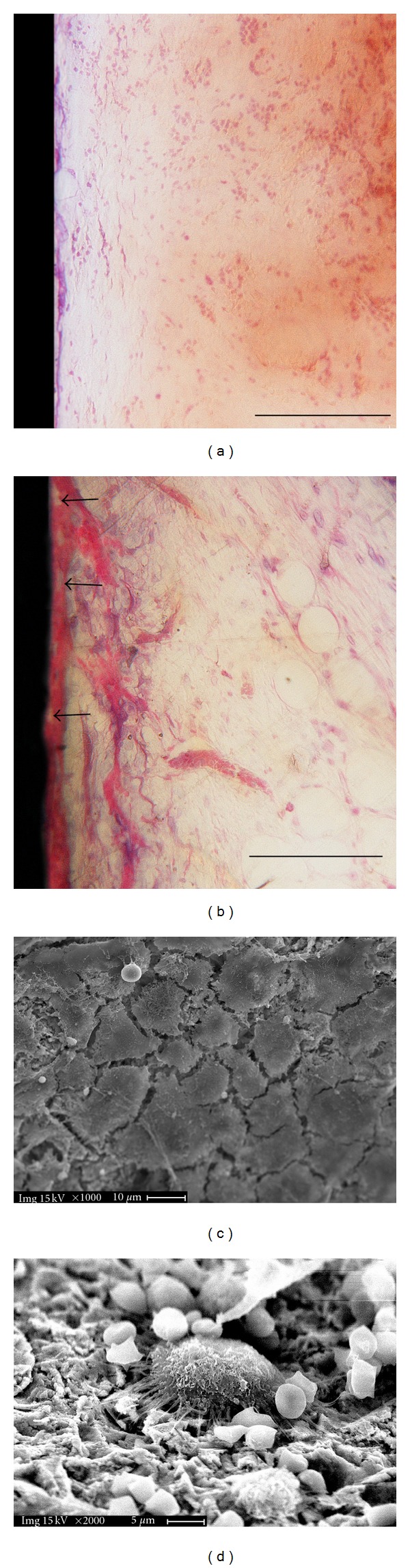
Day 6. Histology of longitudinal ground sections of MS (a) and SLA (b) surfaces. Empty osteocytic lacunae (arrows). LM, toluidine blue, and acid fuchsin staining. Bar = 100 *μ*m. Scanning electron microscopy of deacrylated ground sections of SLA implants: spreaded polygonal osteoblast-like cells (c) and osteoclast-like cell presenting a ruffled border (d) adherent to the metal surface. Since the samples were initially processed for light microscopy (buffered formalin fixative) and afterwards deacrylated for SEM observation, the shrinked appearance of blood cells might be considered as an artifact due to the formalin fixation.

Light microscopy of six day ground sections showed that the number of inflammatory and red blood cells reduced almost completely. The implant cavity along with the marrow spaces were filled by a loose connective matrix with sprouting blood vessels and fat cells. A large number of mesenchymal-like cellular elements with large rounded nuclei appeared to increase in density at the bone-implant interface. In particular, flattened and elongated fibroblast-like cells were present on the perimeter of MS implant surfaces along with some blood vessels forming a continuous layer (Figure 5(a)). By comparison, on SLA surfaces facing the marrow cavity, cuboidal osteoblast-like cells were arranged in parallel to the surface forming a layer next to a darkly stained thin line; these cells appeared more numerous than those on MS (Figure 5(b)). They also appeared to be metabolically active in producing a high pink osteoid rim juxtaposed to the implant, as histologically demonstrated by Toluidine blue-acid fuchsin staining. The same ground sections were therefore deacrylated and the metal surface at the interface was investigated under SEM. Ultrastructural observations confirmed the features of the cells present on the surfaces. In particular, on SLA surfaces, cells showed a cuboidal/polygonal shape and were widely spreaded. They presented cytoplasmatic prolongations by which they came in contact with other cells and with the surface roughness peaks (Figure 5(c)). Some ultrastructural evidences of osteoclast-like cells, such as a ruffled border, could be detected on cells adjacent to the cuboidal ones (Figure 5(d)). 

## 4. Discussion

Bone healing around a titanium dental implant is a complex biological process not yet fully understood. There is a growing interest especially in investigating the very early events taking place at the interface during the healing process. In particular, research focuses on the posterior region of the maxilla which is characterized by a very thin cortical bone shell, an alveolar bone volume reduced in height, and an overall low bone density. This is consistent with the clinical observation that tooth replacement treatments experience the lowest predictability and long-term success in trabecular bone tissue [4]. The proximal tibial epiphysis of rabbit was therefore selected as the experimental animal model because of its anatomical heterogeneity regarded as a representative model for the jaw bony architecture [18]. The aim of the current pilot study was to investigate the early healing events that were triggered by SLA implants inserted in rabbit tibial epiphysis over the period 0–6 days. MS implants were used as control.

Histological and ultrastructural investigations showed a different healing pattern for microrough and machined surfaces since the device insertion step. A few hours after implant placement, a coagulum of blood cells entrapped in a thin network of fibrin was detected at both implant surface types along with primitive marrow cells and inflammatory cells. Many authors have suggested that this immediate interaction with biological microenvironment is governed by surface topography [12, 20, 21]. In the current study, the blood clot appeared to be less dense on SLA and more abundant on MS surfaces where bundles of fibrin-like fibers were arranged in a tight network firmly adherent to the superficial grooves. The peri-implant gap area also contained several chips of host bone, as consequences of surgical drilling and placing procedures, surrounded by blood and marrow elements.

The following events at the bone-implant interface appeared according to a chronological sequence [22]. At day 3, the sequence of morphological events which foreshadow newly peri-implant osteoid deposition occurred earlier at SLA surfaces compared to MS. In particular, at the SLA interface, the presence of a more dense and mature connective stroma, more richly vascolarized and organized, was morphologically evident as long as signs of bone resorption by TRAP-positive osteoclasts present on the trabeculae surfaces. Consequently, some ALP-positive osteoblasts starting the deposition of a new osteoid seam were detected. The new bone formation occurred very early in the first days of wound healing. It started as resorption phenomena around preexisting bone at a distance from the implant, and it proceeded directed toward the implant surface, according to a model known as “distance osteogenesis” [10]. The present authors suggest that the conditioning superficial layer, which appeared less dense on SLA and therefore more easily replaced, could be accountable for this secondary surface cell recruitment pattern. This observation is consistent with data from other researchers [23, 24].

By day 6, the morphological differences between the two surfaces were clear. On SLA surface, it was possible to appreciate a cuboidal osteoblast seam depositing intense-stained osteoid matrix characterized by the presence of large osteocyte lacunae. In contrast, on the MS surface, only a few cuboidal cells could be detected and most of the cells appeared flat and elongated. These findings were consistent with literature which showed how attachment was higher and differentiation faster on microroughness surface in the scale dimension of cell adhesion structures [25, 26]. The new bone formation phenomena, already remarkable at day 3 as distance osteogenesis, was visualized directly in tight contact with some SLA implant areas and raising the possibility of contact osteogenesis. It has been hypothesized that this contact osteogenesis at the implant surface is dependent on a surface-conditioning layer which in turn leads to a specific cell differentiation pattern [18, 27].

From day 6, samples osteoclast-like cells (exhibiting the typical “ruffled border”) were detected at the SLA implant surface. According to other authors [28, 29], osteclast-like cells might be present not only where bone remodeling starts, but in general where osteogenesis occurs as well as, since bone formation process results from osteoblast-osteoclast coupling activity. We speculate that osteoclastic differentiation might be driven by surface topography, according to the geometric tropism of metal peaks and valleys which offer cell anchorage.

In conclusion, the preliminary morphological data hereby described were achieved by means of an experimental *in vivo* model system consisting of a titanium plate-like device with different surfaces (microrough versus machined), inserted in rabbit proximal tibial epiphysis. Because of these device shape and placement site, the most portion of the implant surface lacked direct contact with host bone but faced a wide peri-implant space rich in marrow tissue. This wide peri-implant space was intentionally created in order to dismiss a direct contact between host bone and metal implant and to better understand the role of the biological microenvironment at the interface. Therefore, the authors did not consider peri-implant gap area parameter which is known to play an important role in osseointegration process if not too wide [27, 30, 31]. The study confirmed that the insertion of titanium devices into the proximal tibia elicited a sequence of healing events. Newly formed bone proceeded through an early distance osteogenesis, common to both surfaces, and a delayed contact osteogenesis which seemed to follow different patterns at the two surfaces. In fact, SLA devices showed a more osteoconductive behavior retaining a less dense blood clot, which might be earlier and easier replaced, and leading to a surface-conditioning layer promoting osteogenic cell differentiation and appositional new bone deposition at the titanium surface. This model system is expected to provide a sequence of morphological events useful to clarify the early cellular and biomolecular events occurring at the metal surface.
